# Utility of Chinese Versions of Addenbrooke’s Cognitive Examination: A Narrative Review

**DOI:** 10.3390/healthcare10102052

**Published:** 2022-10-17

**Authors:** Ling-Xiao Cao, Gang Wang, Qi-Hao Guo, Wei Zhang, Thomas Bak, Yue Huang

**Affiliations:** 1 China National Clinical Research Center for Neurological Diseases, Beijing Tiantan Hospital, Capital Medical University, Beijing 100070, China; 2 Department of Neurology, Beijing Tiantan Hospital, Capital Medical University, Beijing 100070, China; 3 Department of Neurology, Institute of Neurology, Ruijin Hospital, School of Medicine, Shanghai Jiao Tong University, Shanghai 200020, China; 4 Department of Gerontology, Shanghai Jiao Tong University Affiliated Sixth People’s Hospital, School of Medicine, Shanghai Jiao Tong University, Shanghai 200233, China; 5 Psychology Department, The University of Edinburgh, Edinburgh EH1 1LT, UK; 6 School of Medical Sciences, Faculty of Medicine, University of New South Wales, Sydney 2033, Australia

**Keywords:** Addenbrooke’s cognitive examination, Chinese, mild cognitive impairment, dementia, clinical examination

## Abstract

Addenbrooke’s cognitive examination (ACE) is a cognitive screening tool that has developed through three stages: ACE, ACE-Revised (ACE-R), and ACE-Ⅲ. In addition, mini-Addenbrooke’s Cognitive Examination (M-ACE) and ACE mobile are the additional versions that is derived from ACE-III. ACE and its related versions show better performance than Mini-Mental State Examination (MMSE) and Montreal Cognitive Assessment (MoCA) in detecting mild cognitive impairment in different neurological disorders. It has been translated into numerous languages, including Chinese. Through reviewing the history, validity, and comparison with other cognitive tests of Chinese versions of ACE, it aims to facilitate the clinical and scientific use, further development, improvement, and validation of Chinese versions of ACE in various neurological disorders and ultimately promote early identification and management of cognitive impairment in China.

## 1. Introduction

Dementia is a major challenge for global public health [1]. Currently, more than 46 million people worldwide suffer from dementia, and it is estimated that this number will increase to 131.5 million by 2050 [2]. As the most populous country in the world, the number of dementia patients in China accounts for approximately 25% of the total number of dementia cases in the world [3]. In China, the prevalence of dementia in people aged over 60 is 4.30–6.30% [4]. Despite the high prevalence of dementia, many people with cognitive impairment are still not correctly diagnosed in a timely manner. It is reported that about 75% of dementia patients worldwide have not been diagnosed, equivalent to 41 million people [5]. Failure to identify dementia earlier poses a great challenge to application of clinical treatment and healthcare. Therefore, the detection method of dementia is particularly important. One of the major methods to detect cognitive impairment is the use of cognitive examination tests. A good cognitive screening tool can help researchers and clinicians identify cognitive impairment early and accurately. So far, apart from Addenbrooke’s Cognitive Examination (ACE), there are many cognitive screening tools, such as the Mini-Mental State Examination (MMSE), Montreal Cognitive Assessment (MoCA), Blessed Dementia Rating Scale (BDRS), and Hasegawa’s Dementia Scale (HDS). ACE and its associated versions have good sensitivity and specificity in terms of detecting cognitive impairment worldwide, and ACE has been proven superior to some of widely used classical scales. Since its introduction to China, ACE has gained acceptance as a valuable tool for cognitive assessment in a variety of neurological conditions. The Chinese versions of ACE, however, are not frequently utilized. Therefore, it is necessary to systematically and historically evaluate different Chinese ACE versions. The review aims to facilitate the clinical and scientific use of Chinese versions of ACE, further development and improvement, and further validation in various neurological disorders, ultimately promoting early identification and management of cognitive impairment in China.

## 2. Search Methodology

This narrative review followed the PRISMA statement. A bibliographic search of studies was conducted until January 2022 using the following key words: “Addenbrooke’s Cognitive Examination, Chinese, China, evaluate, evaluation, validation”. The databases, including PubMed, Google Scholar, and Web of Science, were used for literature searches. Non-English and literature without full-text available were excluded. Articles that used but without evaluation of the Chinese ACEs were excluded. Two reviewers will independently screen the articles. Disagreements between reviewers will be solved by consensus or through the participation of a third reviewer. Following this literature screening standard, nine articles were selected (Supplementary Figure S1). Quality Assessment of Diagnostic Accuracy Studies tool (QUADAS-2) was used to assess the methodological quality of the studies (Supplementary Figure S2).

## 3. History of ACE and Chinese Versions of ACE

**Figure 1:**
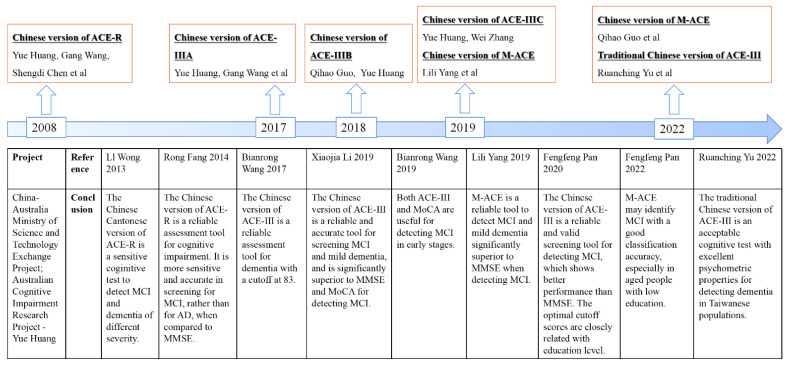
The history of Chinese versions of ACE [23,24,36,37,39,40,42,43,44]. Abbreviations: ACE, Addenbrooke’s Cognitive Examination; ACE-R, Addenbrooke’s Cognitive Examination-Revised; ACE-III, Addenbrooke’s Cognitive Examination III; M-ACE, Mini-Addenbrooke’s Cognitive Examination; MMSE, Mini-Mental State Examination; MoCA, Montreal Cognitive Assessment; MCI, mild cognitive impairment; AD, Alzheimer’s disease.

The development of ACE has gone through three stages: ACE, Addenbrooke’s Cognitive Examination-Revised (ACE-R), and Addenbrooke’s Cognitive Examination III (ACE-III). ACE was developed by Professor John R Hodges in 2000 [6]. It was a novel scale designed to detect mild dementia and distinguish between Alzheimer’s disease (AD) and frontotemporal dementia (FTD). In addition, it was used to detect cognitive impairment in Parkinson’s disease (PD), stroke, and neuropsychiatric diseases, etc. [7,8,9,10]. Although there was no initiation of multilingual use at the beginning, due to its good performance, ACE has been translated into a variety of languages, including Persian, Danish, Spanish, French, and German [9,11,12,13,14,15]. However, to our knowledge, there was no Chinese version of the original ACE. Because of the shortcomings of ACE, such as limited visuospatial component and ceiling effects, and in order to expand the cross-cultural applications, the improved revision, ACE-R, was developed in 2006 [16]. Furthermore, three different alternative versions of ACE-R-A, B, and C were produced to avoid practice effects. ACE-R is revised from ACE to make the stimuli and their interpretation recognizable/common across many cultures. ACE-R is widely used across cultures, including Korean, German, Spanish, Greek, Italian, and Japanese [17,18,19,20,21,22]. The Chinese version of ACE-R was also developed by Professors Yue Huang, Gang Wang, and Sheng-Di Chen in 2008 [23]. The other Chinese version of ACE-R-Chinese-Cantonese was translated specifically for the Hong Kong population in order to increase the practicability of ACE-R in different regions and dialects in China [24]. To overcome the weaknesses of ACE-R, such as poor performance in verbal repetition in healthy adults, translation difficulties, and lacking sensitivity in the comprehension section, ACE-R was modified into ACE-III in 2012 [25]. Since then, ACE-III has become the most widely used version, having been translated into more than 33 languages [26], and it is also considered a good scale for screening cognitive impairment. ACE-III is applied in a wider field of screening cognitive dysfunction in different diseases, including mild cognitive impairment (MCI), AD, FTD, stroke, PD, alcohol-related brain damage, schizophrenia, and so on [25,27,28,29,30,31,32,33,34,35]. The Chinese version of ACE-III was translated in 2012 [36], and its parallel versions were developed in 2018 and 2019 (Figure 1). In addition to the paper-based ACE-III, there are two other versions: a short version called Mini-Addenbrooke’s Cognitive Examination (M-ACE) and an electronic version called ACE mobile (iPad version). M-ACE was translated into Chinese in 2019 [37,38,39]. In 2022, ACE-III was translated into traditional Chinese for use in Taiwanese [40]. ACE mobile is an automated management, guidance, scoring, and reporting tool derived from ACE-III that is free to use on iTunes on iPad for research purposes [41]. As far as we know, there is no translated version of ACE mobile yet.

## 4. Utility of Chinese Versions of ACE in the Detection of Cognitive Impairment

### 4.1. ACE-R

The Chinese version of ACE-R, the same as the original version, consists of five cognitive domains: attention/orientation, memory, fluency, language, and visuospatial. It takes about 12 to 20 min to complete the test, and the total score is 100. There are a few modifications based on the underlying principle during the translation process. For example, the name and address in the memory, recall, and recognition section are replaced by Chinese name and address; the letter ‘P’, which generated as many words as possible, is replaced with Chinese character ‘che, 车’ in the verbal fluency section; English words and sentences are replaced by Chinese characters or poems with difficulties to produce in the repetition section [23].

The Chinese version of ACE-R is a reliable examination test for detecting cognitive impairment, with its satisfactory sensitivity (0.920, 0.867), specificity (0.857, 0.706), and area under curve (AUC) (0.945, 0.836) to detect mild AD and MCI, respectively (Table 1) [23]. The Chinese-Cantonese version of ACE-R is also an excellent cognitive screening tool for MCI and dementia, with acceptable sensitivity (0.74, 0.93), specificity (0.84, 0.95), and AUC (0.84, 0.98) [24]. In the Cantonese speaking Chinese population, ACE-R Cantonese version is recommended, although the majority of Cantonese speaking Chinese can speak Mandarin nowadays. The Chinese version of ACE-R is widely used in the detection of cognitive impairment in amyotrophic lateral sclerosis, multiple system atrophy, PD, primary blepharospasm, and related disorders [42,45,46,47,48,49,50,51]. Three domains (attention, memory, and language) are declined in amyotrophic lateral sclerosis [45]. With the exception of attention, four domains can be affected in patients with multiple system atrophy (MSA), while four domains (except memory) are impaired in PD [48,49]. In addition, all five domains are impaired in primary blepharospasm [50].

### 4.2. ACE-III

More clinicians and medical researchers are using Chinese ACE-III in cognitive assessment, as ACE is gradually modified and improved. Thus, there are more studies using the Chinese version of ACE-III than ACE-R (Table 1) [36,43,44,52]. ACE-III is also scored out of 100 and consists of five domains. Based on specific cultures and usage experiences of ACE-R, the Chinese version of ACE-III has been translated and modified from the original version. For example, the first and third pictures have neem replaced by ‘pencil’ and ‘panda’ in the language domain. The second question in the language domain has been revised to ‘Which animal lives in Sichuan China?’, while Sichuan is a province in China where pandas live. Wang et al. verified ACE-III with satisfactory sensitivity (0.911), specificity (0.831), and AUC (0.952) for detecting dementia [36]. Li et al. and Wang et al. suggested that the Chinese version of ACE-III was a reliable and valid tool for detecting MCI [43,52]. In comparison to studies on the Chinese version of ACE-R, ACE-III shows better performance (Table 1). In addition, the Chinese version of ACE-III is slightly more accurate in participants with ≥12 years of education (AUC = 0.97) than those with <12 years of education (AUC = 0.93) while screening mild dementia [52]. In summary, the Chinese version of ACE-III is a reliable screening tool to detect dementia as well as MCI with different cutoffs [36,43,52]. In Pan et al.’s study, participants were classified as having a low education (1–9 years), a middle education (10–15 years), or a high education (≥16 years). The AUC for ACE-III reached a higher level in the high education subgroup (0.949) than those in the middle education subgroup (0.905) and the low education subgroup (0.894), indicating that the Chinese version of ACE-III performs better for highly educated people [44]. Apart from years of education, age at examination is another factor affecting ACE-III performance [52,53]. However, age at examination is not a strong influencer of ACE-III performance compared to education [44], similar to the findings in the study using ACE-R [16]. In addition to the simplified Chinese version, the traditional Chinese version of ACE-III is also a promising screening tool for detecting dementia in Taiwanese people (AUC = 0.895) [40]. The language spoken at home may influence ACE-III performance, as this phenomenon has been observed for different Indian language versions of ACE-III [54]. Unfortunately, apart from Mandarin and traditional Chinese versions of ACE-III, there are no other Chinese language versions available, such as Tibetan, Mongolian, Cantonese, or Uyghur. Although Mandarin is the official language in China, it may underestimate the cognitive performance for the people of non-Mandarin speaking homes [24]. In addition, premorbid IQ may also influence ACE-III performance [55], which has not been verified in the Chinese version yet.

Similar to ACE-R, aside from detecting early stages of AD, ACE-III has been used for tracking performances of cognitive domains in various neurological disorders [25,27,28,29,30,31,32,33,34,35] (Table 2). Memory, rather than other cognitive domains, is more impaired in AD [25], while fluency and language are more impaired in behavioral variant frontotemporal dementia (bvFTD) and primary progressive aphasia (PPA), respectively [25]. Three domains (attention, memory, and fluency) decline in alcohol-related brain damage, and memory and visuospatial are impaired in rheumatoid arthritis [28,32]. The Hungarian ACE-III is able to delineate cognitive decline in PD with all five domains affected [31]. Memory and fluency domains are impaired in Polish patients with multiple sclerosis (MS), and attention, fluency, language, and visuospatial domains can be affected if there are focal cerebellar lesions [30,34]. Attention, memory, and fluency are impaired in schizophrenia detected by the Thai ACE-III, while memory and visuospatial function are impaired in brain glioma detected by the Malayalam ACE-III [29,35]. Aphasia and other dysfunction induced by stroke are also important factors affecting the accuracy of cognitive tests, which means participants need assistance to complete tests or are unable to complete tests. A modified cutoff can improve diagnostic accuracy, sensitivity, and specificity in stroke patients [33]. So far, the Chinese ACE-III has only been used in AD for cognitive domain analysis, not in other neurological disorders [24].

### 4.3. M-ACE

Due to the wide range of cognitive domains assessed and patients’ cooperation, it usually takes 12 to 20 min to complete the ACE-III test, so the usage of ACE-III may be limited by time constraints in some specific conditions. Thus, M-ACE, a shorter version of ACE-III, was created for this situation in 2015. M-ACE consists of 5 items with a maximum score of 30. The Chinese version of M-ACE is a reliable and quick examination test to detect MCI and mild dementia with its fair sensitivity (0.88, 0.96), specificity (0.72, 0.87), and AUC (0.86, 0.96) (Table 1) [37]. Pan et al. used Chinese version of M-ACE with a total score of 38 to reduce false positive odds and improve the classification accuracy. The Chinese version of M-ACE provides a sensitivity of 0.83, a specificity of 0.80, and an AUC of 0.89 (Table 1). In addition, age and years of education have a significant impact on scores of the Chinese version of M-ACE [37,39], and a better performance (AUC = 0.958) is observed in aged people with low education [39].

## 5. Comparison of Chinese Versions of ACE with Other Screening Techniques

### 5.1. ACE-R

Compared with the MMSE, the Chinese version of ACE-R has a higher sensitivity and AUC to screen for MCI (Table 1) [23], which is consistent with other studies using different linguistic ACE-R versions [19,56]. However, the AUC value of the Chinese ACE-R for detecting mild AD is not as good as the MMSE [23], which is consistent with a study using the German ACE-R [19]. This is in contrast to the majority of other studies showing that ACE-R is superior to the MMSE in detecting dementia [17,19,57]. This may be due to the small sample size or fewer years of education of the study cohorts [23]. The study of the Chinese–Cantonese version of ACE-R shows that it is a sensitive and specific cognitive screening test, and it is similar to the MMSE in identifying MCI (0.84 for sensitivity, 0.85 for specificity) and dementia (0.98 for sensitivity, 0.98 for specificity) [24]. Thus, in the Cantonese speaking Chinese population, the ACE-R Cantonese version is recommended, despite the fact that the majority of Cantonese speaking Chinese can speak Mandarin nowadays. A meta-analysis was conducted by Huo et al. to evaluate the diagnostic accuracy of the Chinese versions of dementia screening tools in the Chinese population [58]. One hundred and thirty-four studies including 81 screening tools in Chinese were applied in this meta-analysis. According to this study, the MMSE was the most commonly used cognitive screening scale, while the Chinese version of the ACE-R showed the best performance with the highest sensitivity (0.96) and specificity (0.96) [58].

### 5.2. ACE-III

Unlike the MMSE, which is unidimensional and provides a global deterioration of intellect, ACE-III is multidimensional and can be scored independently according to its five components: attention/orientation, memory, language, verbal fluency (executive functions), and visuospatial skills to generate a cognitive profile (Table 3).

The verbal fluency of ACE-III provides good evaluation value for assessing frontal lobe function. ACE-III shows fewer ceiling effects and better performance in detecting MCI than the MMSE [43,52], similar to the studies using other linguistic versions of ACE-III [59,60,61]. Consistent with studies using other linguistic versions of ACE-III or ACE-R [54,62], participants with longer years of education (≥12 years) have a better performance on Chinese version ACE-III compared to the MMSE (AUC 0.97 vs. 0.90), whereas Chinese version ACE-III does not perform better than MMSE in detecting dementia in lower-educated participants (<12 years) (AUC 0.93 vs. 0.98) (Table 1) [52]. ACE-III is designed with more comprehensive domains and more challenging tasks compared with MMSE, while MMSE has a very strong impact on orientation and languages. Thus, for memory, the most affected cognitive domain of amnestic MCI, it accounts for a reasonable proportion in ACE-III. The Chinese version of ACE-III is either equivalent to or significant superior to MoCA in detecting MCI in different studies [43,44,52]. Other studies using different linguistic versions of ACE-III demonstrated a higher diagnostic accuracy of ACE-III for distinguishing MCI than MoCA [59,63,64].

### 5.3. M-ACE

The Chinese version of M-ACE appears to have a better performance in detecting MCI and mild dementia than the MMSE with higher sensitivity, specificity, and accuracy (Table 1) [37,39]. The results are consistent with other studies using different linguistic versions of M-ACE [38,59,65,66,67]. The M-ACE is also proven to be more sensitive and have less ceiling effect than MMSE. Studies from Japan and Greece showed that their linguistic versions of M-ACE were superior to MoCA in detecting MCI and dementia [59,68], but studies using the English or Chinese versions of M-ACE did not reach the same conclusion [39,69]. In addition, the Chinese version of M-ACE also shows comparable accuracy to the Chinese version of ACE-III (AUC 0.892 vs. 0.901) [39].

## 6. Discussion

All Chinese versions of ACE-R, ACE-III, and M-ACE have been proven reliable, sensitive, and valid in cognitive screening. In addition, the latest Chinese version of ACE-III and its shorter version, M-ACE, have been proven to be superior to the MMSE and MoCA, the most widely used cognitive screening tools, especially in individuals with higher education. ACE-III has been proven to be superior to ACE-R, and it is more widely used than previous versions in medical research. In addition, previous studies using the Chinese ACE-III for cognitive screening are mostly monocentric studies, and a large multicenter study is required to further validate the efficacy of ACE-III in different geographic regions of China with different dialects. In addition, the Chinese ACE-III has not been widely used in clinics, and the parallel Chinese versions are not fully verified. Furthermore, ACE-III is freely available for medical research, whereas other screening tools may be restricted by copyright (such as MMSE and MoCA). At present, the Chinese version of ACE-III is recommended by the Chinese Medical Association in the white paper ‘Standardized Protocol for diagnosis and treatment of cognitive impairment’ as a cognitive screening tool in 2021 [70]. Currently, the MMSE is still a preferable cognitive screening tool for many clinicians in China, despite its weakness in detecting MCI. Thus, a conversion table between the original version of ACE-III and MMSE has been developed [43,71]. A conversion table between the Chinese version of ACE-III and MMSE is waiting to be developed. In addition, the English version of ACE-III has been used to detect the differences in cognitive domains in different neurological disorders, but there is no such a kind of study using the Chinese version of ACE-III.

Finally, mobile ACE is a pragmatic tool with its convenience, automation, easy storage, and management. In the clinical usage of paper-based ACE-III, 78% of the usage had either incorrect answers or arithmetical errors, and mobile ACE can reduce the errors by 85–93% [41]. Therefore, the Chinese version of mobile ACE is worthwhile to be developed and used in China in the future.

There are several limitations of this review. We excluded studies applying the Chinese version of ACE without systematic evaluation, as there were no raw data available, which reduced the number of studies reviewed. In addition, there is no electronic version of the Chinese ACE-III, making the Chinese ACE versions incomplete in relation to English versions.

## 7. Conclusions

To decide which ACE version is conducted, there are several factors to be considered: for research purposes, ACE-III is widely used for comprehensive cognitive assessment, and M-ACE is most likely to be used in busy clinics due to time constraints. Although the latest Chinese version of ACE-III is recommended, it needs to be translated into multi-ethnic language versions and applied in multiple regions, with comparable other cognitive scales to further verify its effectiveness in different neurological disorders. Furthermore, the Chinese electronic version of ACE-III needs to be developed and promoted to be widely used in China. We hope that the wide application of ACE will promote early identification and management of cognitive impairment in China in the future.

## Figures and Tables

**Table 1:** Published literature using Chinese versions of ACE.

Reference	Cognitive Impairment Status		Cognitive Measures	Cutoff Score	Sensitivity	Specificity	AUC
Fang et al., 2013 [23]	MCI		ACE-R	85/86	0.867	0.706	0.836
			MMSE	27/28	0.520	0.863	0.751
	Mild AD		ACE-R	67/68	0.920	0.857	0.945
			MMSE	23/24	1.000	0.937	0.996
Wong et al., 2014 [24]	MCI		ACE-R C	79/80	0.74	0.84	0.84
			MMSE	26/27	0.76	0.81	0.85
	Dementia		ACE-R C	73/74	0.93	0.95	0.98
			MMSE	25/26	0.96	0.88	0.98
Wang et al., 2017 [36]	Dementia		ACE-III	83	0.911	0.831	0.952
			MMSE	NA	NA	NA	0.827
Li et al., 2019 [52]	MCI		ACE-III	88/89	0.75	0.89	0.88
			MMSE	28/29	0.64	0.63	0.72
			MoCA	24/25	0.67	0.77	0.76
	Mild dementia		ACE-III	74/75	0.94	0.83	0.95
			MMSE	25/26	0.89	0.71	0.95
			MoCA	21/22	0.88	0.93	0.91
Wang et al., 2019 [43]	MCI		ACE-III	85	0.973	0.907	0.978
			MMSE	28	0.838	0.817	0.891
			MoCA	23	0.978	0.875	0.965
Pan et al., 2021 [44]	MCI	Low education (1–9)	ACE-III	72	0.806	0.830	0.894
			MMSE	27	0.776	0.648	0.763
			MoCA	23	0.857	0.818	0.899
		Middle education (10–15)	ACE-III	78	0.823	0.832	0.905
			MMSE	27	0.654	0.739	0.765
			MoCA	24	0.869	0.824	0.913
		High education (≥16)	ACE-III	80	0.839	0.867	0.949
			MMSE	27	0.714	0.819	0.816
			MoCA	24	0.875	0.857	0.946
Yang et al., 2019 [37]	MCI		M-ACE	25/26	0.88	0.72	0.86
			MMSE	27/28	0.82	0.44	0.69
	Mild dementia		M-ACE	21/22	0.96	0.87	0.96
			MMSE	25/26	0.88	0.87	0.94
Pan et al., 2022 [39]	MCI		M-ACE	25	0.830	0.800	0.892
			ACE-III	77	0.811	0.824	0.901
			MMSE	27	0.701	0.740	0.782
			MoCA	23	0.824	0.875	0.916
Yu et al., 2022 [40]	Dementia		T-ACE-III	73/74	0.895	1.000	0.895

Abbreviations: ACE, Addenbrooke’s Cognitive Examination; AUC, area under curve; ACE-R, Addenbrooke’s Cognitive Examination-Revised; ACE-R C, Addenbrooke’s Cognitive Examination-Revised Cantonese version; ACE-III, Addenbrooke’s Cognitive Examination III; M-ACE, Mini-Addenbrooke’s Cognitive Examination; T-ACE-III, Taiwanese ACE-III; MMSE, Mini-Mental State Examination; MoCA, Montreal Cognitive Assessment; MCI, mild cognitive impairment; AD, Alzheimer’s disease; NA, not available.

**Table 2:** Cognitive domain decline tested by ACE-III for neurological disorders.

Neurological Disorders		Language	Changes in Cognitive Domains					Reference
			Attention	Memory	Fluency	Language	Visuospatial	
AD		English	*	**	*	*	*	[25]
FTD	bvFTD	English	*	*	**	*	*	[25]
	PPA	English	*	*	*	**	NC	[25]
PD		Hungarian	*	*	*	*	*	[31]
MS		Polish	NC	*	*	NC	NC	[34]
Schizophrenia		Thai	*	*	*	NC	NC	[29]
ARBD		English	*	*	*	NC	NC	[28]
FCL		Polish	*	NC	*	*	*	[30]
Brain tumor		Malayalam	NC	*	NC	NC	*	[35]
RA		English	NC	*	NC	NC	*	[32]

* Significant declined; ** The most affected. Abbreviations: AD, Alzheimer’s disease; FTD, frontotemporal dementia; bvFTD, behavioral variant frontotemporal dementia; PPA, primary progressive aphasia; PD, Parkinson’s disease; MS, multiple sclerosis; ARBD, alcohol-related brain damage; FCL, focal cerebellar lesions; RA, rheumatoid arthritis; NC, not changed compared to that of controls.

**Table 3:** Differences among ACE-III, M-ACE, MMSE, and MoCA.

Cognitive Scale	Domain	Total Score	Time	Advantages	Disadvantages
ACE-III	Attention, memory, fluency, language, and visuospatial	100	15 to 20 min	Best sensitivity and specificity, better performance in highly educated population	Time consuming
M-ACE	Orientation, memory, language, and visuospatial	30	5 min	Time saving, better performance than MMSE and MoCA	Insufficient evidence in other diseases
MMSE	Orientation, attention, memory, and language	30	5 to 10 min	Time saving, not requiring high level of education for patients	Uneven scores in different domains, ceiling effect, and insensitivity in detecting MCI
MoCA	Orientation, attention, language, visuospatial, memory, and executive	30	10 to 15 min	Extensive domains, sensitivity in detecting MCI	Time consuming, insensitivity in low level of education for patients

Abbreviations: ACE-III, Addenbrooke’s Cognitive Examination III; M-ACE, Mini-Addenbrooke’s Cognitive Examination; MMSE, Mini-Mental State Examination; MoCA, Montreal Cognitive Assessment; MCI, mild cognitive impairment.

## Data Availability

Not applicable.

## Supplementary Materials

The following supporting information can be downloaded at: https://www.mdpi.com/article/10.3390/healthcare10102052/s1, Figure S1: Flow chart of literature search for this review; Figure S2: Quality assessments of included studies.
