# Supplementary material for: Utility of Chinese Versions of Addenbrooke’s Cognitive Examination: A Narrative Review
Source: Healthcare (Basel). 2022 Oct 17;10(10):2052. doi: 10.3390/healthcare10102052 (PMC9602941; doi:10.3390/healthcare10102052)
Supplement: Supplementary file 1 [file healthcare-10-02052-s001.zip › healthcare-1966177-supplementary.pdf]

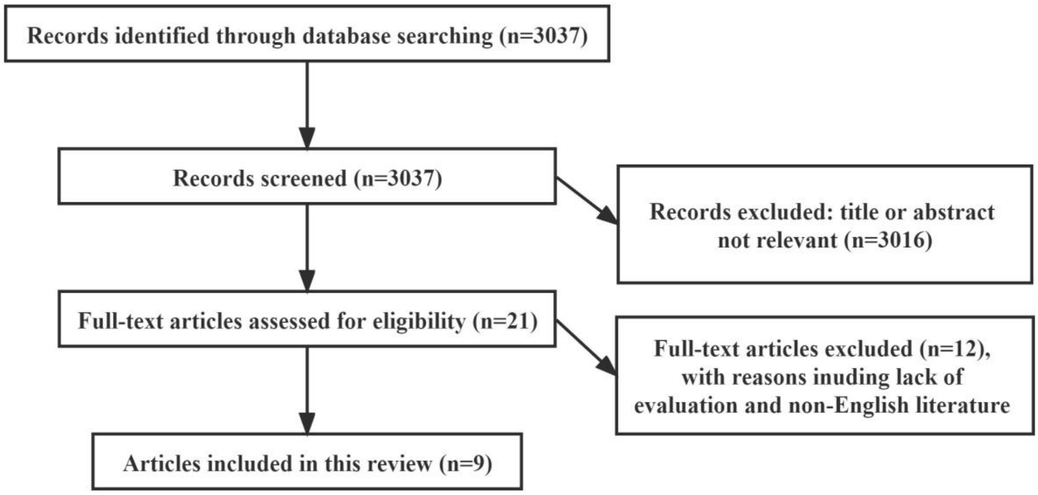

Supplementary Figure S1. Flow chart of literature search for this review.
This review followed the PRISMA statement. Keywords for searching include "Addenbrooke's Cognitive Examination, Chinese, China, evaluate, evaluation, and validation".

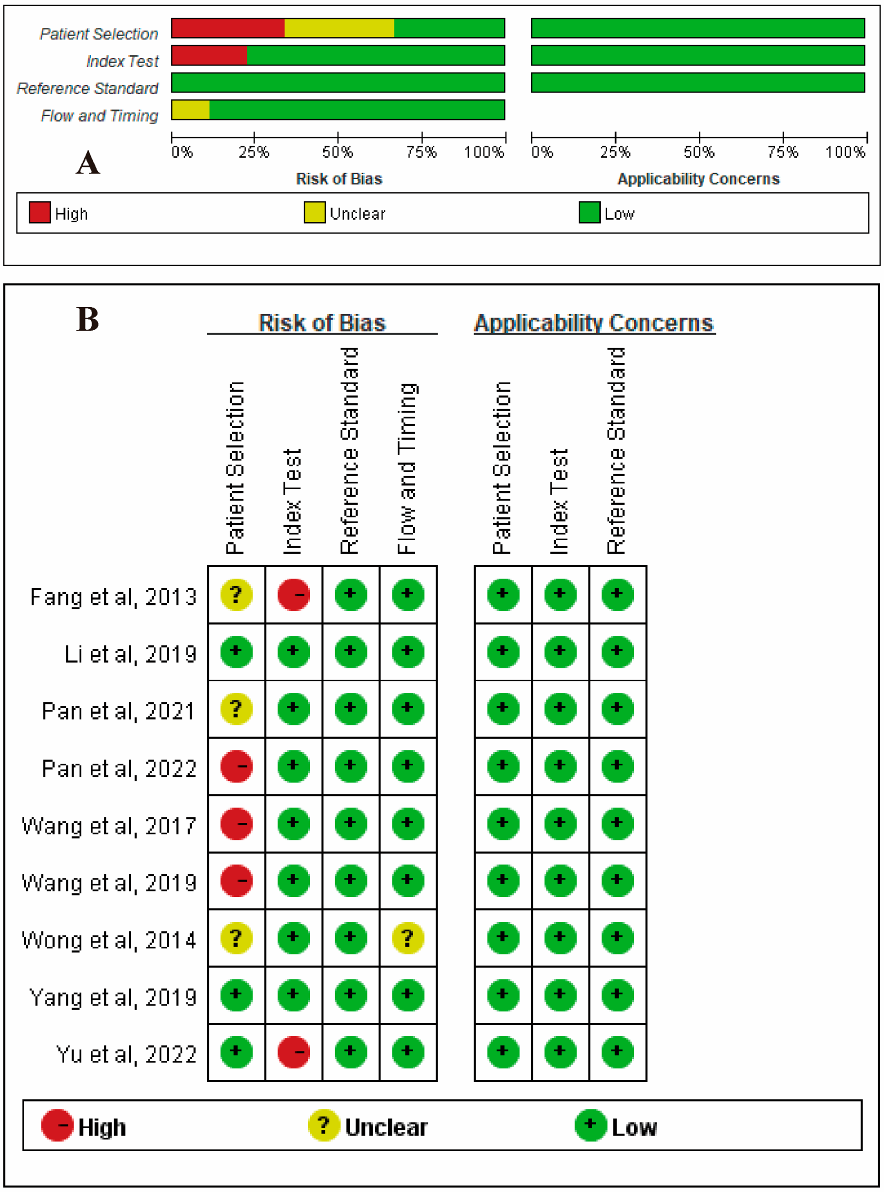

Supplementary Figure S2. Quality assessments of included studies.

A, risk of bias and applicability concerns graph; B, risk of bias and applicability concerns summary.
